# Sources and Transmission Routes of Carbapenem-Resistant *Pseudomonas aeruginosa*: Study Design and Methodology of the SAMPAN Study

**DOI:** 10.3390/antibiotics14010094

**Published:** 2025-01-15

**Authors:** Anneloes van Veen, Selvi N. Shahab, Amber Rijfkogel, Anne F. Voor in ’t holt, Corné H. W. Klaassen, Margreet C. Vos, Yulia Rosa Saharman, Anis Karuniawati, Silvia Zelli, Desy De Lorenzis, Giulia Menchinelli, Giulia De Angelis, Maurizio Sanguinetti, Merel Kemper, Anniek E. E. de Jong, Sima Mohammadi, Valentine Renaud, Irena Kukavica-Ibrulj, Marianne Potvin, Guillaume Q. Nguyen, Jeff Gauthier, Roger C. Levesque, Heike Schmitt, Juliëtte A. Severin

**Affiliations:** 1 Department of Medical Microbiology and Infectious Diseases, Erasmus MC University Medical Center, 3015 GD Rotterdam, The Netherlands; a.vanveen@erasmusmc.nl (A.v.V.); s.shahab@erasmusmc.nl (S.N.S.); a.rijfkogel@erasmusmc.nl (A.R.); a.voorintholt@erasmusmc.nl (A.F.V.i.’t.h.); c.h.w.klaassen@erasmusmc.nl (C.H.W.K.); m.vos@erasmusmc.nl (M.C.V.); 2 Department of Clinical Microbiology, Faculty of Medicine, Universitas Indonesia, Dr. Cipto Mangunkusumo General Hospital, Jakarta 10430, Indonesia; yulia.rosa01@ui.ac.id (Y.R.S.); anis.karuniawatimk@ui.ac.id (A.K.); 3 Dipartimento di Scienze di Laboratorio e Infettivologiche, Fondazione Policlinico Universitario Agostino Gemelli IRCCS, 00168 Roma, Italy; silvia.zelli@guest.policlinicogemelli.it (S.Z.); desy.delorenzis@guest.policlinicogemelli.it (D.D.L.); giulia.menchinelli@policlinicogemelli.it (G.M.); giulia.deangelis@unicatt.it (G.D.A.); maurizio.sanguinetti@policlinicogemelli.it (M.S.); 4 Centre for Infectious Disease Control, National Institute for Public Health and the Environment (RIVM), 3720 BA Bilthoven, The Netherlandsheike.schmitt@rivm.nl (H.S.); 5 Deltares, 2629 HV Delft, The Netherlands; anniek.dejong@deltares.nl; 6 Institut de Biologie Intégrative et des Systèmes (IBIS), Université Laval, Quebec City, QC G1V 0A6, Canada; sima.mohammadi.1@ulaval.ca (S.M.); valentine.renaud.1@ulaval.ca (V.R.); kukavica@fmed.ulaval.ca (I.K.-I.); marianne.potvin@qo.ulaval.ca (M.P.); guillaume-quang-henri.nguyen.1@ulaval.ca (G.Q.N.); jeff.gauthier.1@ulaval.ca (J.G.); rclevesq@ibis.ulaval.ca (R.C.L.); 7 Department of Biotechnology, Technical University Delft, 2628 CD Delft, The Netherlands

**Keywords:** antimicrobial drug resistance, carbapenems, hospitals, humans, One Health, *Pseudomonas aeruginosa*, risk factors, wastewater

## Abstract

**Background/Objectives**: The global spread of carbapenem-resistant *Pseudomonas aeruginosa* (CRPA) warrants collaborative action. Guidance should come from integrated One Health surveillance; however, a surveillance strategy is currently unavailable due to insufficient knowledge on the sources and transmission routes of CRPA. The aim of the SAMPAN study (“A Smart Surveillance Strategy for Carbapenem-resistant *Pseudomonas aeruginosa*”) is to develop a globally applicable surveillance strategy. **Methods**: First, an international cross-sectional study will be conducted to investigate CRPA in clinical and environmental settings in Rotterdam (The Netherlands), Rome (Italy), and Jakarta (Indonesia). Screening cultures and risk factor questionnaires will be taken from healthy individuals and patients upon hospital admission. Clinical CRPA isolates will also be included. Additionally, samples will be taken twice from wet hospital environments and monthly from the hospitals’ (drinking) water system, hospital and municipal wastewater treatment plants, and receiving rivers. Whole-genome sequencing will be performed to characterize CRPA isolates and determine the genetic relatedness among the isolates from different reservoirs. Findings from the cross-sectional study, combined with expert elicitation using a Delphi method, will serve as the input for the surveillance strategy. **Conclusions**: The SAMPAN study will provide a broader understanding of the sources and transmission routes of CRPA. Therewith, the development of a globally applicable smart surveillance strategy will be made possible, delivering information that is needed to guide actions against the spread of CRPA.

## 1. Introduction

Antimicrobial resistance (AMR) is an increasing global health challenge. In 2019, 1.27 million deaths worldwide were attributable to AMR, a number expected to increase to about 10 million annual deaths by 2050 [1,2]. One of the leading pathogens responsible for these deaths due to AMR is *Pseudomonas aeruginosa* [1]. Especially, infections with carbapenem-resistant *P. aeruginosa* (CRPA) are difficult to treat, cause a high disease burden, and are associated with an all-cause mortality rate of 30% and higher [1,3,4,5]. Consequently, the World Health Organization (WHO) has classified CRPA as a high-priority pathogen in their 2024 list, requiring urgent attention and action [3].

To design and focus such action, information from an integrated global surveillance strategy following a transdisciplinary One Health approach is needed, as antimicrobial-resistant bacteria do not only arise in and spread among humans. An example of such an integrated surveillance strategy that has proven to be useful is the WHO Tricycle protocol for extended-spectrum beta-lactamase (ESBL)-producing *Escherichia coli* [6,7,8]. However, it is likely that the sources and transmission routes are different for CRPA compared to ESBL-producing *E. coli*, which may require an emphasis on other key elements of such a surveillance strategy.

The human–water interface plays a fundamental role in the spread of CRPA, as these bacteria prefer moist environments. Inside of hospitals, transmission of CRPA from wet environmental niches, such as faucets, sinks, and showers, to hospitalized patients can occur and is estimated to account for over 85% of transmission events [9,10,11]. Patients colonized or infected with CRPA may, in turn, cause persistent environmental contamination in the hospital setting and/or transmission to other patients. Most of the reports on the environmental reservoirs and transmission pathways of CRPA inside of hospitals are derived from outbreak settings in high-income countries [9]. However, the role of environmental sources in the spread of CRPA in non-outbreak and low-resource settings is largely unknown.

**Figure 1:**
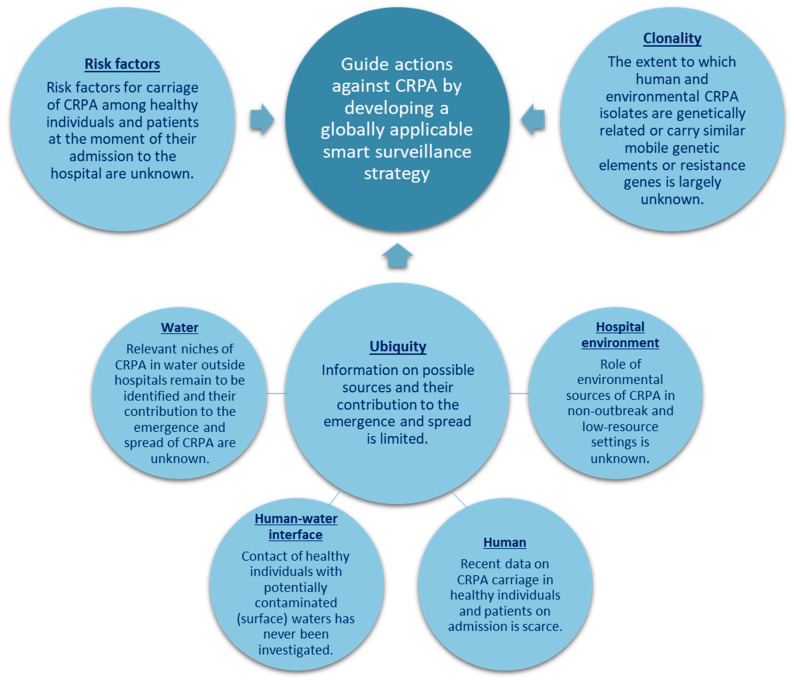
Knowledge gaps addressed in the SAMPAN study. CRPA: carbapenem-resistant *Pseudomonas aeruginosa*.

Through human feces, CRPA may disseminate from the treated or untreated effluent of hospital or municipal wastewater treatment plants (hWWTPs/mWWTPs) into the aquatic environment outside of hospitals, including rivers, lakes, and coastal waters [12,13,14]. Also, the horizontal transfer of carbapenem resistance genes may further promote the development and spread of CRPA in sewage systems and receiving waters. Consequentially, the aquatic environment may function as a source of CRPA spread and poses a threat to public health, especially when sewage effluents are discharged in the environment untreated and humans depend on these receiving waters for drinking, washing, and sanitation [15]. Data on the carriage rates of *P. aeruginosa* in healthy individuals range from 1.5% to 15.8%, yet recent data on CRPA in healthy individuals and risk factors are lacking [16,17,18,19,20,21]. Also, to what extent contaminated (surface) waters contribute to the spread of CRPA in humans, and to what extent human and environmental isolates are genetically related and carry similar resistance genes and/or mobile genetic elements, is largely unknown. While various sources and transmission routes of CRPA, both inside and outside of hospitals, have been identified, the development of an integrated global surveillance strategy is hindered by multiple knowledge gaps (Figure 1).

**Figure 2:**
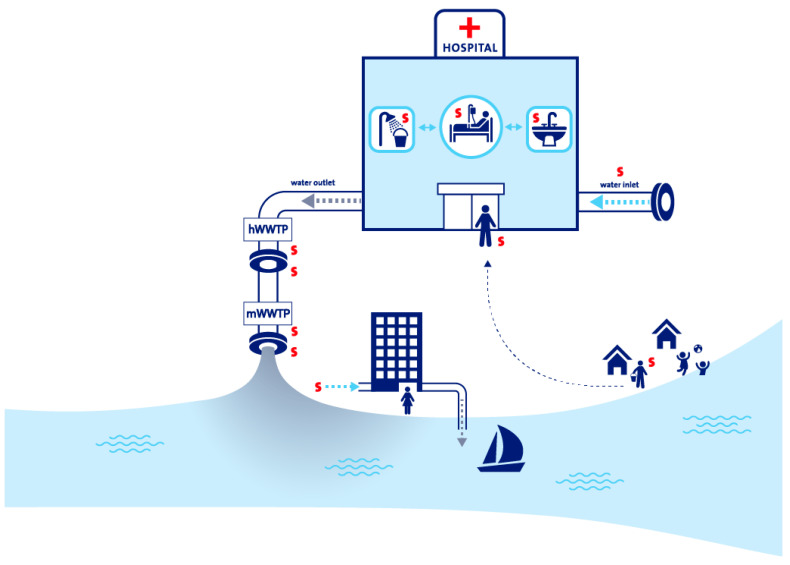
Overview of the human–water interface investigated in the SAMPAN study. hWWTP: hospital wastewater treatment plant; mWWTP: municipal wastewater treatment plant. The red “S” indicates the sampling sites.

In the SAMPAN study (“A Smart Surveillance Strategy for Carbapenem-resistant *Pseudomonas aeruginosa*”), we aim to develop a globally applicable smart surveillance strategy to guide actions against the spread of CRPA. Prior to formulating such a strategy, an international cross-sectional One Health study will be conducted to investigate the carriage rates, risk factors for carriage, and relevant sources and transmission routes of CRPA in different settings, with a particular emphasis on the human–water interface (Figure 2).

The specific aims of the international cross-sectional One Health study are as follows:

1. Determine the carriage rates of CRPA and the risk factors for carriage in healthy individuals and patients at the moment of their admission to the hospital.
2. Gain insight into the potentially relevant niches of CRPA in the moist environments of hospitals in different settings (non-outbreak, and high- and low-resource settings), as well as in (surface) water inside and outside of hospitals in three cities with different climates and water management systems.
3. Unravel the genetic relatedness of CRPA in terms of clonality, resistance genes, and mobile genetic elements within the different reservoirs and climates in order to confirm possible transmission routes.

Subsequently, the findings of the international cross-sectional One Health study, together with expert opinion, will be used as the input in a Delphi method to develop the globally applicable smart surveillance strategy for CRPA.

## 2. Materials and Methods

### 2.1. Study Design and Setting

**Figure 3:**
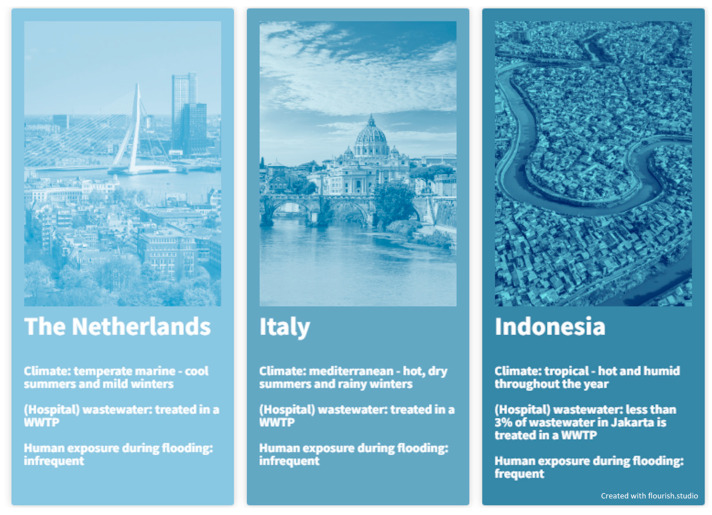
Overview of the climate and water situations in the Netherlands, Italy, and Indonesia. WWTP: wastewater treatment plant.

The initial phase of the SAMPAN study involves conducting a multicenter, cross-sectional One Health study. In this study, we will investigate the carriage rates, risk factors for carriage, and relevant sources and transmission routes of CRPA in humans and the environment. The study will take place in the following three countries: the Netherlands, Italy, and Indonesia. The selection of these countries is based on differences in climate, water management approaches, and the anticipated CRPA prevalence, thereby, providing the opportunity to study the sources and transmission routes of CRPA in various settings (Figure 3).

One hospital per country will be involved in this study (Table 1), including the Erasmus MC University Medical Center (Rotterdam, The Netherlands), Fondazione Policlinico Universitario Agostino Gemelli IRCCS (Rome, Italy), and Dr. Cipto Mangunkusumo General Hospital (Jakarta, Indonesia). Samples will be collected from a variety of reservoirs in each city during a period of 12 months, including from healthy individuals, patients upon their admission to the hospital, clinical isolates, wet hospital environments, and water from each hospital’s (drinking) water supply system, hWWTP(s) and mWWTP, and the receiving river. The Medical Ethics Review Committee of each participating hospital gave written approval to conduct the study. This study was registered in ClinicalTrials.gov (registration number: NCT05282082).

### 2.2. Study Population and Recruitment Process

Firstly, we will enroll healthy individuals (i.e., not hospitalized) in each city over a period of one year. We aim to reach a total of 500 participants; however, formal sample size calculations cannot be performed due to the unknown CRPA prevalence in healthy individuals. To determine which healthy individuals to approach for study participation, certain “high-risk areas” were first mapped out for each city. A “high-risk area” is defined as an area that is expected to potentially have a high prevalence of CRPA which may affect humans because of being immediately downstream of the sewage systems of hWWTPs and/or mWWTPs. All of the healthy adult individuals living in these “high-risk areas” are eligible for inclusion in the study and can only participate once throughout the study period.

At first, individuals living closest to the river (<50 m) will be invited to participate either by a home visit from a member of the research team or by receiving a letter in the mailbox, followed by individuals living further away (<100 m). In case this method appears insufficient to recruit healthy individuals, the area will be expanded and different recruitment strategies will be explored, such as using social media, local newspapers, radio stations, and/or other live events to request healthy individuals to participate. Expanding the distance between residential addresses and the river may impact the results; however, whether there will be an effect, and what kind of effect, is currently unknown. We hypothesize that individuals residing nearest to the river may face a higher risk of becoming colonized by CRPA if the river is contaminated with CRPA and if these individuals (frequently) interact with the contaminated river.

Secondly, we aim to enroll 500 hospitalized patients in each city over a period of one year. Similarly, the lack of prevalence data among newly hospitalized patients prevents accurate sample size determination. Patients will be asked to participate within the first 48 h of their admission to the hospital. All of the patients from non-critical medical and surgical wards are eligible for inclusion if they are aged 18 years or older, if they have an expected length of stay of at least 24 h, if they can provide answers to a questionnaire verbally or in writing, and if they have a signed informed consent sheet. Patients will only be included once during the inclusion period. Patients that are unwilling to provide a rectal/perianal swab and patients with cystic fibrosis (CF) are excluded from study participation. The enrolment progress of healthy individuals and newly hospitalized patients will be monitored closely by the central study coordinators (A.v.V. and J.S.) in order to ensure the highest possible number of participants.

Clinical CRPA isolates from patients throughout the entire hospital, as identified through routine diagnostics, will also be collected. Only one CRPA isolate per patient will be included. If multiple CRPA isolates are available from the same patient, the first isolate will be included. If more than one isolate is available from that same date, the most resistant CRPA isolate (i.e., the highest MIC and/or resistance to a wider range of antibiotics) will be included. Clinical CRPA isolates from CF patients are not eligible for inclusion.

### 2.3. Data Collection

#### 2.3.1. Human Samples

Table 2 provides an overview of the methods that will be used in each country to collect different types of samples. Healthy individuals will be asked to self-sample and/or to have throat, navel, and rectal/perianal swabs taken once after providing informed consent. Self-sampling kits, which can be send to healthy individuals’ homes, contain an information letter, step-by-step instructions (including images and links to online videos), and self-sampling materials to assist in taking the required swabs correctly [22]. Healthy individuals can provide the swabs immediately during a home visit or live event, or return the swabs by regular mail. For healthy individuals, the distance of each individual’s home to the water sampling locations will be calculated by a Python script using the distance between the centric point of the zip code area to the mWWTP, and river upstream and downstream sampling locations.

After obtaining informed consent, throat, navel, and rectal/perianal swabs will be taken once from the patients within the first 48 h of their admission. Patients will also be allowed to self-sample after receiving verbal instructions from a member of the research team. Clinical CRPA isolates will be collected in the routine diagnostics laboratories of each hospital. Additionally, basic patient characteristics and admission information will be collected from the (electronic) health records of all patients (including the patients from whom the clinical CRPA isolates originate), such as age at admission, sex, and type of ward. All of the human data will be collected and stored on the online platform Castor, with the metadata presented in Supplementary Table S1.

#### 2.3.2. Questionnaire

A questionnaire was developed by a subgroup of the SAMPAN Consortium, including a clinical microbiologist and an epidemiologist. Questions related to known risk factors of CRPA carriage during hospitalization (e.g., the presence of underlying diseases, antibiotic use, having medical devices, etc.) are based on a study by Voor in ‘t holt et al. [23], and questions related to drinking water and sanitation are based on a guidance document by the WHO and the United Nations Children’s Fund (UNICEF) [24]. In total, the questionnaire contains 37 questions, and consists of 6 general questions, 10 questions related to the participant’s health, 3 questions related to international travel, and 18 questions related to contact with water. The questionnaire was developed in English and was subsequently translated into Dutch, Italian, and Indonesian, and is only available on paper for the participants. Healthy individuals and patients, and, when possible, patients from whom the clinical CRPA isolates are collected, will receive the same questionnaire (with the options for different languages) (Supplementary File S2).

#### 2.3.3. Environmental Samples

The hospital environment will be sampled twice throughout the study period, once at the beginning and once at the end of the sampling year. Samples will be collected from the sink drains and shower drains in all of the patient rooms, including their attached (private) bathrooms, from the wards in which the patients will be enrolled for study participation. Additional sampling sites were chosen per hospital based on potential ‘high-risk’ reservoirs for CRPA. Supplementary File S1 (Figures S1–S3) provides an overview of the sampling sites in each hospital. The sampling methods described by Van der Schoor et al. will be used to collect the samples [25]. In brief, a sterile cotton swab will be pre-moistened using phosphate-buffered saline (PBS). For the sink drains, the cotton swab will be inserted 1–3 cm inside of the drain, followed by sampling the inner surface of the drain pipe while rotating the swab. In the case where a plug covering the sink drain is present, the swab will be inserted underneath the sink plug to sample the inner sink environment. The outer surface of the shower drain will be sampled in multiple directions while rotating the swab and using overlapping motions.

Water samples will be taken once per month from a variety of sampling sites (Table 2). Inside of the hospitals, the sampling sites include the (drinking) water inlet, and the influent and effluent of the hWWTP(s) (where existing), where the raw sewage from the hospital is collected and treated before discharge into the receiving river (Indonesia) or into the municipal sewer network leading to the mWWTP (The Netherlands). Outside of the hospitals, the samples will be collected from the influent and effluent of the receiving mWWTP (The Netherlands and Italy) or an mWWTP in proximity (Indonesia), as well as river upstream and downstream of the discharge point of the hWWTPs/mWWTP. Different methods will be used to collect water samples depending on the accessibility of the sampling site. Grab samples will be taken from, e.g., a tap point for the (drinking) water inlet, a basin, and river upstream and downstream of the hWWTPs/mWWTP, with the latter by the use of a telescope pole with an attached beaker. Sampling will follow the standardized EN ISO 16266 methods [26]. In the Netherlands, 24-h flow proportional samples using automated samplers will be taken from the influent of the hWWTP, and from the influent and effluent of the mWWTP. Supplementary File S1 (Figures S4–S9) provides an overview of the sampling locations in each city as well as schematics of the hospital wastewater treatment systems. Samples will be collected by researchers from the study group and/or staff from the mWWTPs. Subsequently, water samples will be transported to the laboratory, while stored at 4 °C, on the day of sample collection and analyzed within 24 h.

### 2.4. Microbiological Methods

#### 2.4.1. Samples from Healthy Individuals and Patients upon Hospital Admission

The culture methods, as described in Shahab et al. [27,28], will be used for the samples from healthy individuals and patients upon hospital admission. In short, rayon swabs (Thermo Scientific™ Oxoid™ Amies Agar Gel Transport Swabs, ThermoFisher Scientific, Breda, The Netherlands) will be used to collect throat, navel, or rectal/perianal swabs. The swabs will be enriched in Tryptic Soy Broth (TSB) with 2 mg/L vancomycin and 2 mg/L imipenem as selective agents by adding disks to the broth, followed by overnight incubation at 35 °C. Vancomycin is added to the broth to suppress the growth of Gram-positive bacteria. The choice of imipenem as a selective agent is based on MIC considerations, with imipenem being more often resistant than meropenem in important CRPA clones, as well as the finding that imipenem in disks is more stable than other carbapenems [27,29,30]. Subsequently, 10 µL of the broth will be inoculated onto a *Pseudomonas*-selective agar, M-PA-C agar (BD Diagnostics, Breda, The Netherlands), and incubated at 35 °C for 18–24 and 48 h.

#### 2.4.2. Hospital Environment Samples

Sterile cotton swabs will be used to collect samples from the wet hospital environment and, after sample collection, will be enriched in TSB with 2 mg/L vancomycin and 2 mg/L imipenem. After overnight incubation at 35 °C, 10 µL of the broth will be inoculated onto M-PA-C agar, followed by incubation at 35 °C for 18–24 and 48 h.

#### 2.4.3. Water Samples

For the isolation and quantification of CRPA from the water samples, a most probable number (MPN) method will be used, based on filtrations of the water samples. The filtered volumes used in each sampling site are shown in Supplementary Table S2, including a range of volumes with both expectedly negative and expectedly positive results as required for the MPN method. The methods to detect CRPA in water samples have previously been described [31]. Briefly, water samples will be filtered through 0.45 µm pore size membrane filters (Millipore, Burlington, MA, USA). The filters will then be placed in 10 mL Asparagine Proline (ASP) broth (Millipore, Burlington, MA, USA) with 2 mg/L vancomycin, followed by incubation for 18–24 h at 37 °C. After incubation, 10 µL of the enrichment broth will be inoculated onto M-PA-C agar supplemented with 8 mg/L imipenem. After incubation for 18–24 h at 37 °C, presumptive *P. aeruginosa* colonies will be streaked onto the M-PA-C agar and again incubated for 18–24 h at 37 °C. The number of colonies picked is based on the observed number of colony morphologies on the plates, as detailed in Supplementary Table S3. Colonies growing on the M-PA-C agar will then be streaked onto Columbia agar with sheep blood (Oxoid, Basingstoke, UK) and incubated for 18–24 h at 37 °C.

#### 2.4.4. Protocol Alignment

Sampling and laboratory protocols, including instruction videos, will be shared with all members of the research team before the start of enrolment to ensure the standardization of the sampling and culture methods across the three countries. Monthly meetings will be held to discuss the study progress and resolve any emerging issues.

#### 2.4.5. Identification and Antimicrobial Susceptibility Testing

In Jakarta, preliminary identification will be performed by an oxidase test, followed by the identification of all oxidase-positive isolates, using the Vitek^®^2 with a GN Identification Card (bioMérieux, Marcy L’Etoile, France). In Rotterdam and Rome, species-level identification will be performed on all presumptive *P. aeruginosa* colonies using Matrix-Assisted Laser Desorption Ionization–Time-of-Flight Mass Spectrometry (MALDI-TOF MS [Bruker Daltonics, Bremen, Germany]). Antibiotic susceptibility tests will be performed by the Vitek^®^2 with the AST-GN Card in all three cities. However, the AST-GN Card available in Indonesia does not include imipenem in the panel; therefore, disk diffusion for imipenem and meropenem will also be performed in Jakarta. Susceptibility test results will be interpreted according to the Clinical & Laboratory Standards Institute (CLSI) guidelines [32]. CRPA was defined as resistance to at least one of the carbapenems tested (i.e., MIC ≥ 8 for imipenem and/or meropenem). All of the CRPA isolates will be stored at −80 °C prior to further processing.

#### 2.4.6. Genotypic Characterization

(i) Bacterial growth conditions: All of the CRPA isolates will be shipped to the Institut de Biologie Intégrative et des Systèmes (IBIS), Université Laval, Quebec, Canada, for whole-genome sequencing. Briefly, each isolate will be grown by three-step streaking on Tryptic Soy Agar (BD Difco) for 24 h at 37 °C. Then, a pure colony will be grown in Tryptic Soy Broth (BD Difco) for 24 h at 37 °C. The bacterial density will be adjusted to an OD_600nm_ of ~1.0 prior to the DNA extraction.
(ii) Genomic DNA extraction: An amount of 1 mL of liquid culture will be centrifuged at 14,000× *g* for 10 min, and then processed with the QIAGEN DNeasy Blood & Tissue Kit using the manufacturer’s recommended protocol for Gram-negative bacteria. The resulting DNA will be quantified with the Qubit dsDNA BR assay (Thermo Fisher).
(iii) Whole-genome sequencing: An amount of 400 ng of genomic DNA will be sequenced on the Oxford Nanopore GridION platform (Oxford Nanopore Technologies, Oxford, UK) using SQK-LSK114 chemistry on R10.4.1 flow cells. In parallel, 500 ng genomic DNA will be sequenced on the Illumina MiSeq (with TruSeq 3 chemistry) (Illumina, San Diego, CA, USA) to obtain short high-quality reads to correct the assembly gaps and nucleotide mismatch errors. Unless specified otherwise, the manufacturers’ recommended protocols will be followed for either the Nanopore or Illumina library preparation kits [33,34]. Hybrid assemblies will be analyzed for the presence of plasmids, resistance genes, etc., using the appropriate pipelines (the details of which will be included in subsequent manuscripts).

#### 2.4.7. Statistical Analysis

Analyses will be performed using IBM SPSS Statistics (Version 28.0.1.0) and R software (Version 4.3.2), with MPN calculations performed by using the R package ‘MPN’. For descriptive purposes, continuous variables will be presented as means with standard deviations or medians with range, and categorical variables will be presented as absolute numbers with percentages. Risk factors and protective factors for CRPA carriage will be determined using (logistic) regression analysis on different patient characteristics. Factors will be included in the model based on expert opinion and as suggested by the univariate analysis. The number of variables which can be selected for the model depends on the number of CRPA cases found. Sex-based as well as seasonal analyses will be performed. Possible interactions will be explored by comparing models with and without interaction terms using the likelihood ratio test. Non-linear effects exploration will be performed if continuous variables are included. A two-sided *p*-value of <0.05 will be considered statistically significant.

### 2.5. Delphi Method: Development of Globally Applicable Smart Surveillance Strategy for CRPA

Findings from the international cross-sectional One Health study will serve as input for the development of the globally applicable smart surveillance strategy using a Delphi approach. At first, a questionnaire will be developed by members of the SAMPAN study group, including questions to determine the relevance of surveilling different reservoirs, the frequency and methods of sampling, and the outcome measures per reservoir. A large international group of experts (i.e., multidisciplinary, evenly distributed across age groups and sex, and representing a diverse range of countries, evenly distributed across high-income and low- and middle-income countries (LMICs), so to ensure a broad global perspective) will be invited to fill out the questionnaire and evaluate the findings from the international cross-sectional One Health study. The results will be used to develop a draft surveillance strategy, followed by multiple discussion rounds with the group of experts, until consensus on the surveillance strategy is reached. The surveillance strategy that is to be developed should be smart, in the sense that it should adhere to a modified version of the commonly used SMART criteria, ensuring it is specific (i.e., targeting specific target populations and surveillance sites using sound methodologies and metrics), measurable (i.e., with clearly defined outcome measures for each reservoir), achievable (i.e., achievable within a given time period and setting), realistic (i.e., the strategy should be realistic to achieve, also in low-resource settings), and time-efficient (i.e., the execution of the strategy should be time-efficient) [35].

## 3. Discussion

The SAMPAN study is designed to provide insight into the sources and transmission routes of CRPA by conducting an international cross-sectional One Health study and, based on the findings found therein, as well as expert elicitation, to propose a globally applicable smart surveillance strategy. Although several sources and transmission routes of CRPA have been described in the literature, to date, there has been no study investigating the large-scale interactions between humans, including both healthy individuals and patients, and the environment inside and outside of hospitals, including wet environmental niches inside of hospitals and (surface) water. Also, an integrated global surveillance strategy for CRPA is currently not available due to a lack of knowledge on the sources and dynamics of CRPA.

Several One Health surveillance systems have been established, primarily focusing on zoonotic diseases and, increasingly, on AMR [36]. Most existing One Health surveillance systems connect the human and animal sectors, but lack the interlinkage with and monitoring of the environment [36]. The WHO Tricycle protocol for global surveillance on ESBL-producing *E. coli* is the most comprehensive One Health surveillance strategy to date, providing detailed guidance on surveillance in the human, animal, and environmental sectors [6]. Scientific evidence on prevalence rates, including geographic and temporal trends, sources, and cross-sectoral transmission dynamics, led an international group of experts to select and develop the Tricycle protocol with ESBL-positive *E. coli* as an indicator microorganism [6]. With a higher disease burden due to the lack of alternative treatment options and its confirmed presence in the environment, CRPA is an important next target for integrated One Health surveillance [3,14,37]. However, further understanding of its sources and transmission routes is needed as an initial step.

The multicenter design of the international cross-sectional One Health study with three sampling sites in different countries with anticipated differences in the prevalence rates of CRPA, and different climates and water situations, enables us to investigate the sources and transmission dynamics of CRPA in a variety of settings. The sources and transmission routes identified by this study will, therefore, also be relevant for many other countries around the world. Additionally, CRPA will be studied in clinical and environmental settings for the duration of one year, allowing us to examine the seasonality of CRPA carriage, the concentrations of CRPA in (waste)water, and CRPA transmission. Also, the extensive questionnaire used for healthy individuals, patients, and, when possible, for patients from whom clinical CRPA isolates are collected will identify potential risk factors for CRPA carriage. Furthermore, the in-depth genomic analyses using the latest technological advances will offer us the means to explore the molecular epidemiology of CRPA across the three cities, and will identify known and potentially also new genes encoding for carbapenem resistance. Finally, developing a surveillance strategy involves making trade-offs between comprehensiveness (i.e., including all relevant reservoirs, frequent sample collection, etc.) and efficiency in terms of time and cost. The findings of the international cross-sectional One Health study will provide indications as to which reservoirs are the most relevant for effective and efficient surveillance. Combined with expert opinion from across the globe, and building upon recommendations from the EPI-Net One Health consensus working group on multisectoral AMR surveillance reporting, as well as recommendations on developing One Health surveillance systems from the One Health High-Level Expert Panel, this will provide us the opportunity to formulate a surveillance strategy for CRPA that is both globally applicable and smart [38,39]. 

This study has limitations. Even though a variety of settings will be studied in the One Health study, including high-income countries and LMIC, as well as different climatic zones, countries in the African, North American, South American, and Oceanian continents are not included in this study. Also, potential biases may occur during this cross-sectional study, e.g., non-response bias due to the voluntary recruitment of participants. In particular, the recruitment of healthy individuals may be affected by the socioeconomic status of individuals living in the selected “high-risk” areas, with a shift towards the participation of more affluent individuals. Yet, information letters and instructions for self-sampling are provided in simple language and are available in a variety of different languages, if required, to reduce the risk of non-response bias.

In conclusion, the SAMPAN study will provide a broader understanding on the ubiquity and transmission dynamics of CRPA in and between humans and the environment, filling in pre-defined knowledge gaps. As a result, this study will enable us to develop a globally applicable smart surveillance strategy and to identify (a range of) measures that can help to prevent the further spread of CRPA.

## Figures and Tables

**Table 1:** Characteristics of the study sites ^1^.

Characteristics	Erasmus MC University Medical Center, Rotterdam, The Netherlands	Fondazione Policlinico Universitario Agostino Gemelli IRCCS, Rome, Italy	Dr. Cipto Mangunkusumo General Hospital, Jakarta, Indonesia
No. of hospital beds	581	1526	811
Type of patient rooms	100% adult single-occupancy rooms	Multiple-occupancy rooms (mainly two-person rooms)	Multiple-occupancy rooms (mainly more than four-person rooms)
No. of admissions per year	30,288	60,000	13,500
Distance to the nearby river ^2^	“Nieuwe Maas” river: 655 m	“Tevere” river: 2260 m	“Ciliwung” river: less than 10 m
Building area	203,000 m^2^	233,236 m^2^	182,856 m^2^
Surface area	100,000 m^2^	201,066 m^2^	121,926 m^2^
No. of city inhabitants (millions)	0.652	2.872	11.249
Population density (people/km^2^)	3043	2232	14,464

^1^ Numbers reported are from 2022. ^2^ Measured as the shortest distance from the hospital to the nearby river.

**Table 2:** Description of methods used to collect different types of samples per country.

	The Netherlands: Rotterdam Erasmus MC University Medical Center	Italy: Rome Fondazione Policlinico Universitario Agostino Gemelli IRCCS	Indonesia: Jakarta Dr. Cipto Mangunkusumo General Hospital
**Patients at the moment of their admission to the hospital**			
Wards for recruiting patients	Gastroenterology Gynecology Internal medicine Neurosurgery Surgery Urology	Gastroenterology Infectious diseases	Cardiology Geriatric medicine Internal medicine Neurology Surgery
**Clinical CRPA isolates**			
Collection of clinical CRPA isolates	From the entire hospital (except children)	From the entire hospital (except children)	From the entire hospital (including children)
**Healthy individuals**			
Methods used to recruit healthy individuals	Letter in the mailbox Large-scale requests through social media, local newspapers, and local radio stations	Live events	Home visits by members of the research team
Returning swabs to the hospital	Via regular mail	Provided during live events	Provided during home visit
**Hospital environment**			
Sampling sites	Two sink drains and one shower drain per patient room	One sink drain, one shower drain, and one shower head per patient room Sink drain in disabled bathroom Sink drain in kitchen	One sink drain and one shower drain per patient room Sink drains in all nurse stations
**Water**			
Sampling sites	Inside the hospital: Drinking water inlet (tap point) Influent of hWWTP (24-h flow-proportional automated sampler) Effluent of hWWTP (grab sample) Outside the hospital: Influent of mWWTP (24-h flow-proportional automated samplers; 4 influent strands from different city districts) Effluent of mWWTP (24-h flow-proportional automated sampler) River upstream of mWWTP (grab sample) River downstream of mWWTP (grab sample)	Inside the hospital: Water inlet (tap point) Hospital effluent (grab sample) Outside the hospital: Influent of mWWTP (grab sample) Effluent of mWWTP (grab sample) River upstream of mWWTP (grab sample) River downstream of mWWTP (grab sample)	Inside the hospital: Hospital ground water Hospital tap water (tap point) Influent of hWWTPs (grab samples) Effluent of hWWTPs (grab samples) Outside the hospital: Influent of mWWTP (grab sample) Effluent of mWWTP (grab sample) River upstream of hWWTPs (grab sample) River in proximity of hWWTP outlet (grab sample) River downstream of hWWTPs (grab sample) Community ground water (tap point)

CRPA: carbapenem-resistant *Pseudomonas aeruginosa*; hWWTP: hospital wastewater treatment plant; mWWTP: municipal wastewater treatment plant.

## Data Availability

Metadata for human data will be uploaded in the data repository DataverseNL (https://dataverse.nl/dataverse/ErasmusMC), and raw reads, assembly data, and metadata will be uploaded on the International Pseudomonas Consortium Database (https://ipcd.ibis.ulaval.ca). Raw data will be made available by the authors for reuse upon request.

## Supplementary Materials

The following supporting information can be downloaded at: https://www.mdpi.com/article/10.3390/antibiotics14010094/s1, Supplementary File S1: Metadata, sampling locations and water systems. Table S1: Metadata for human data that will be stored in the online platform Castor; Figure S1: Environmental sampling sites in the hospital in Rotterdam, the Netherlands; Figure S2: Environmental sampling sites in the hospital in Rome, Italy; Figure S3: Environmental sampling sites in the hospital in Jakarta, Indonesia; Figure S4: Water sampling sites outside of the hospital in Rotterdam, the Netherlands; Figure S5: Water sampling sites outside of the hospital in Rome, Italy; Figure S6: Water sampling sites outside of the hospital in Jakarta, Indonesia; Figure S7: Wastewater treatment system of the hospital in Rotterdam, the Netherlands; Figure S8: Wastewater treatment system of the hospital in Rome, Italy; Figure S9: Wastewater treatment system of the hospital in Jakarta, Indonesia; Table S2: Filtered volumes per sampling site for the isolation and quantification of CRPA; Table S3: Number of colonies picked based on the observed number of colony morphologies; Supplementary File S2: Questionnaire for the international SAMPAN study. Ref. [40] is cited in the Supplementary Materials.
