# Supplementary material for: Sources and Transmission Routes of Carbapenem-Resistant Pseudomonas aeruginosa: Study Design and Methodology of the SAMPAN Study
Source: Antibiotics (Basel). 2025 Jan 15;14(1):94. doi: 10.3390/antibiotics14010094 (PMC11763197; doi:10.3390/antibiotics14010094)
Supplement: Supplementary file 1 [file antibiotics-14-00094-s001.zip › Supplementary file S1.pdf]

Supplementary file S1: Metadata, sampling locations and water systems

Table S1 Metadata for human data that will be stored in the online platform Castor

Healthy individuals	Patients upon hospital admission	Patients with clinical samples
1. Country	1. Country	1. Country
2. Sex	2. Sex	2. Sex
3. Age	3. Age	3. Age
4. Date of informed consent	4. Date of informed consent	4. Date of informed consent
5. Home's location (i.e. upstream or downstream) relative to the discharge point of the mWWTP (Rotterdam and Rome)/hWWTPs (Jakarta)	5. Date of admission	5. Date of admission
6. Distances of the person's home to the various water sampling locations	6. Ward of admission	6. Ward of admission
7. Microbiological data	7. Specialism at admission	7. Specialism at admission
8. Questionnaire answers	8. Type of patient room	8. Type of patient room
	9. Microbiological data	9. Specimen type
	10. Questionnaire answers	10. Microbiological data
		11. Questionnaire answers

hWWTP hospital wastewater treatment plant, *mWWTP* municipal wastewater treatment plant

Figures S1 Environmental sampling sites in the hospital in Rotterdam, the Netherlands

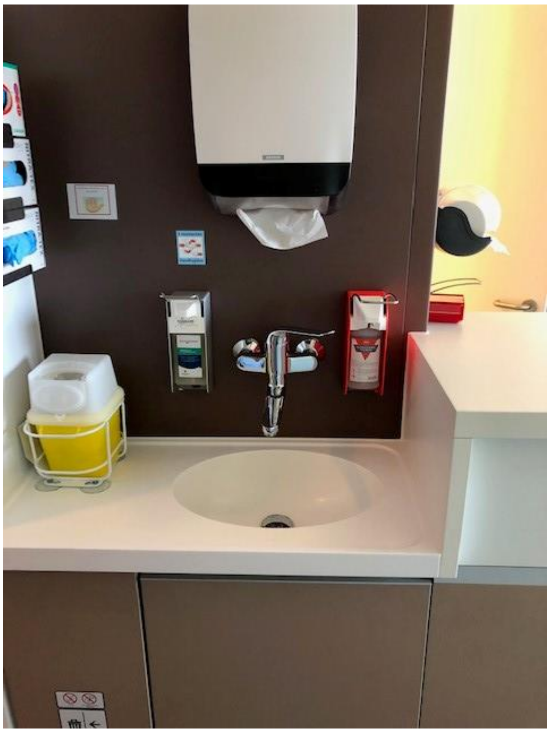

Figure S1A Sink in a single-occupancy room

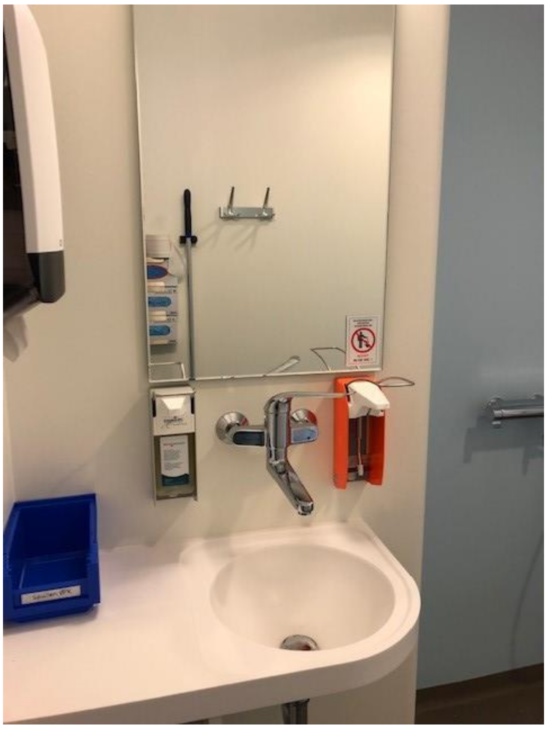

Figure S1B Sink in the attached, private bathroom

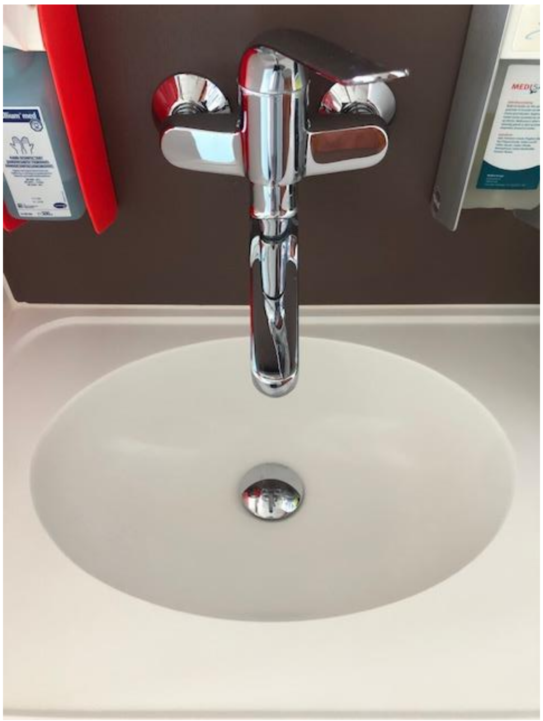

Figure S1C Sink plug

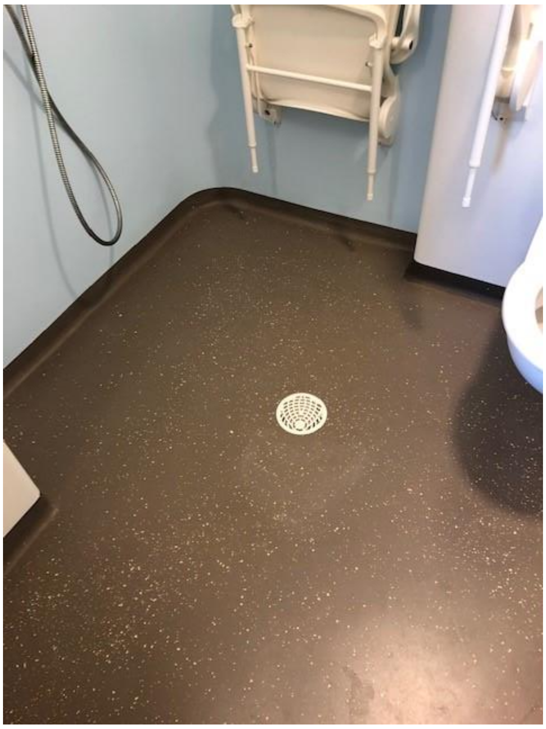

Figure S1D Shower drain in the attached, private bathroom

Figures S2 Environmental sampling sites in the hospital in Rome, Italy

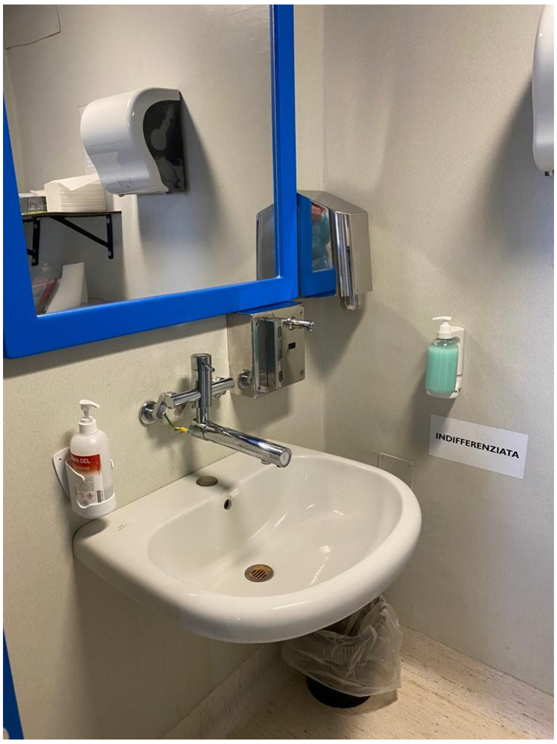

Figure S2A Sink in a double-occupancy room

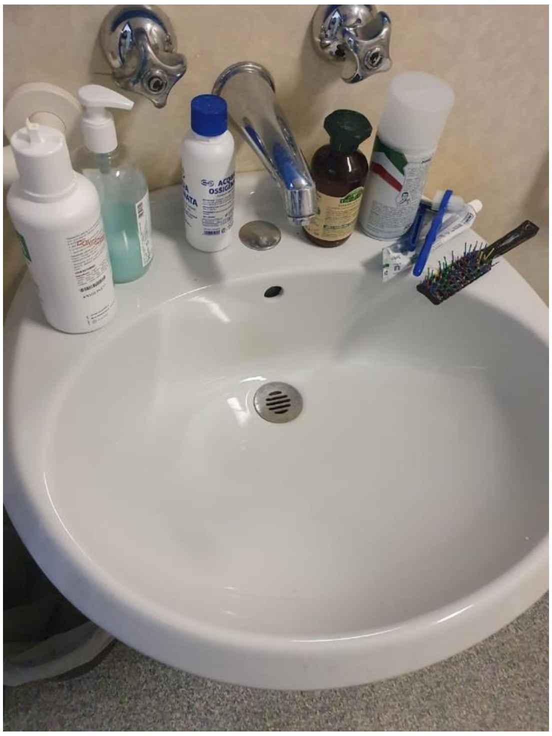

Figure S2B Sink in the attached bathroom

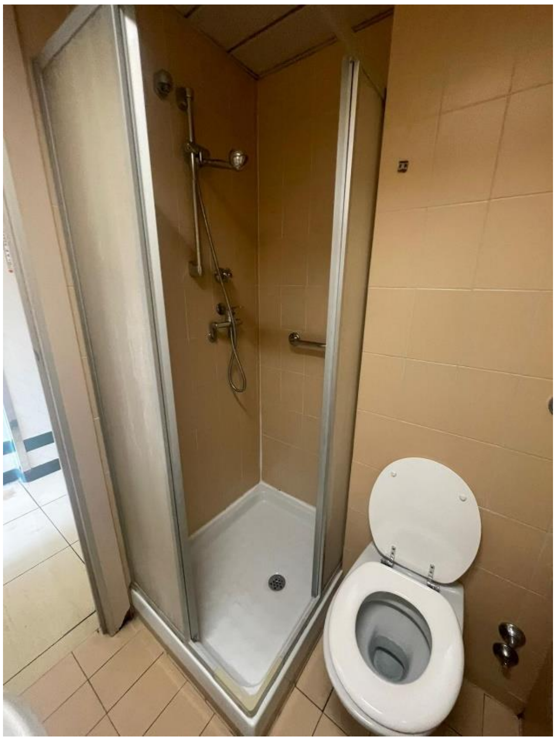

Figure S2C Shower head[1] and shower drain in the attached bathroom

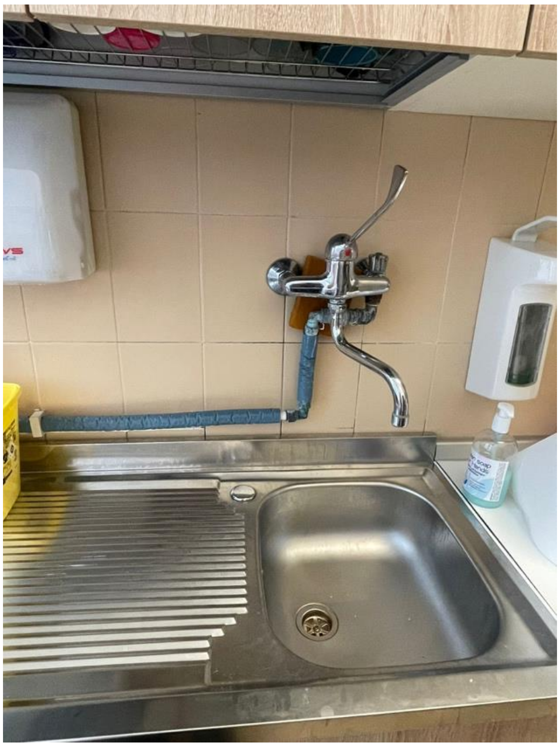

Figure S2D Sink in the kitchen used by healthcare workers

[1] The shower head was sampled by rotating the swab over the entire surface area, while focusing specifically on the shower head nozzles.

Figures S3 Environmental sampling sites in the hospital in Jakarta, Indonesia

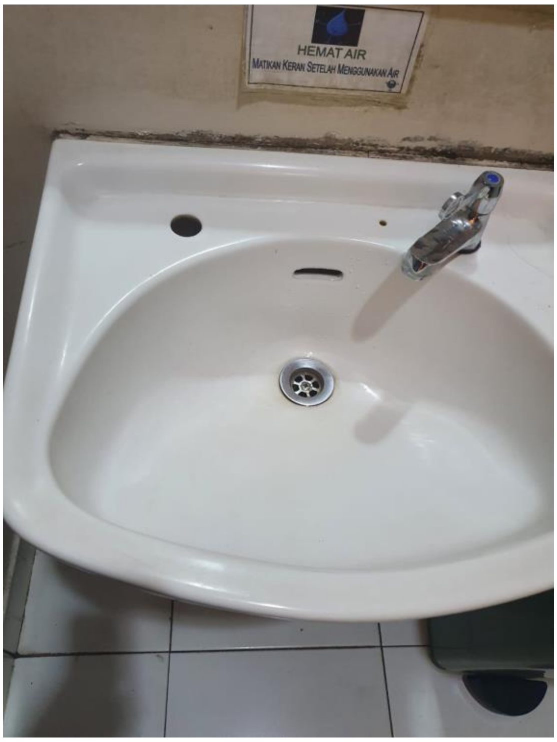

Figure S3A Sink in a multiple-occupancy room

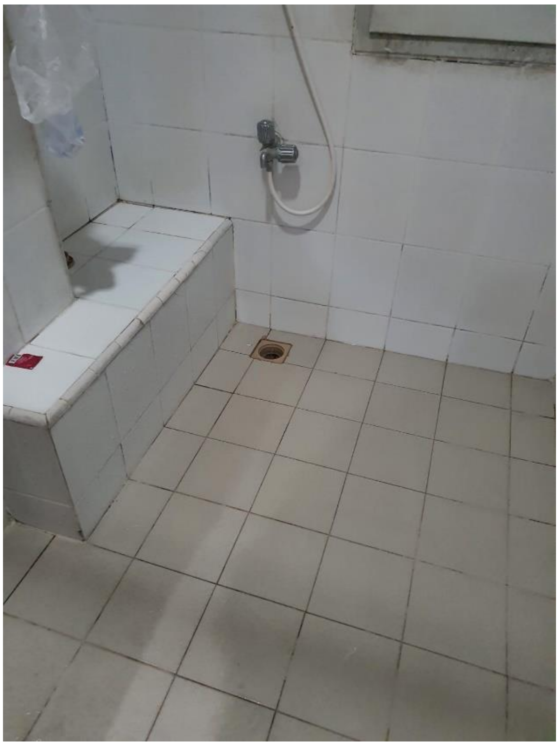

Figure S3B Shower drain in attached bathroom

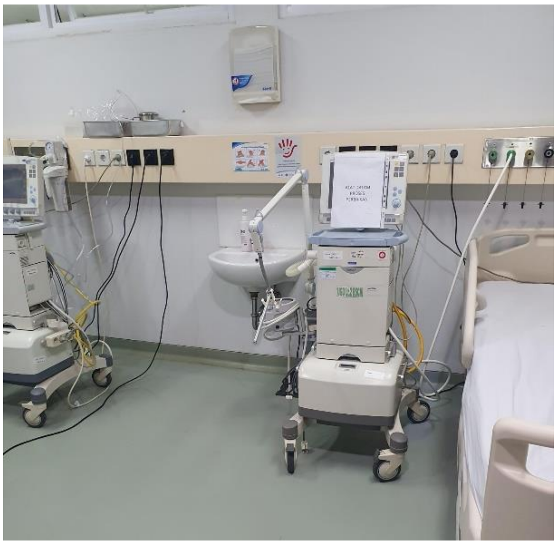

Figure S3C Sink in the adult ICU

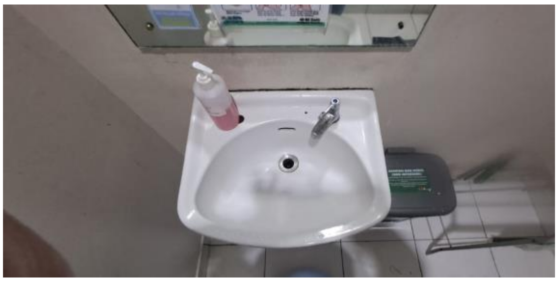

Figure S3D Sink in the adult ICU

Figure S4 Water sampling sites outside the hospital in Rotterdam, the Netherlands

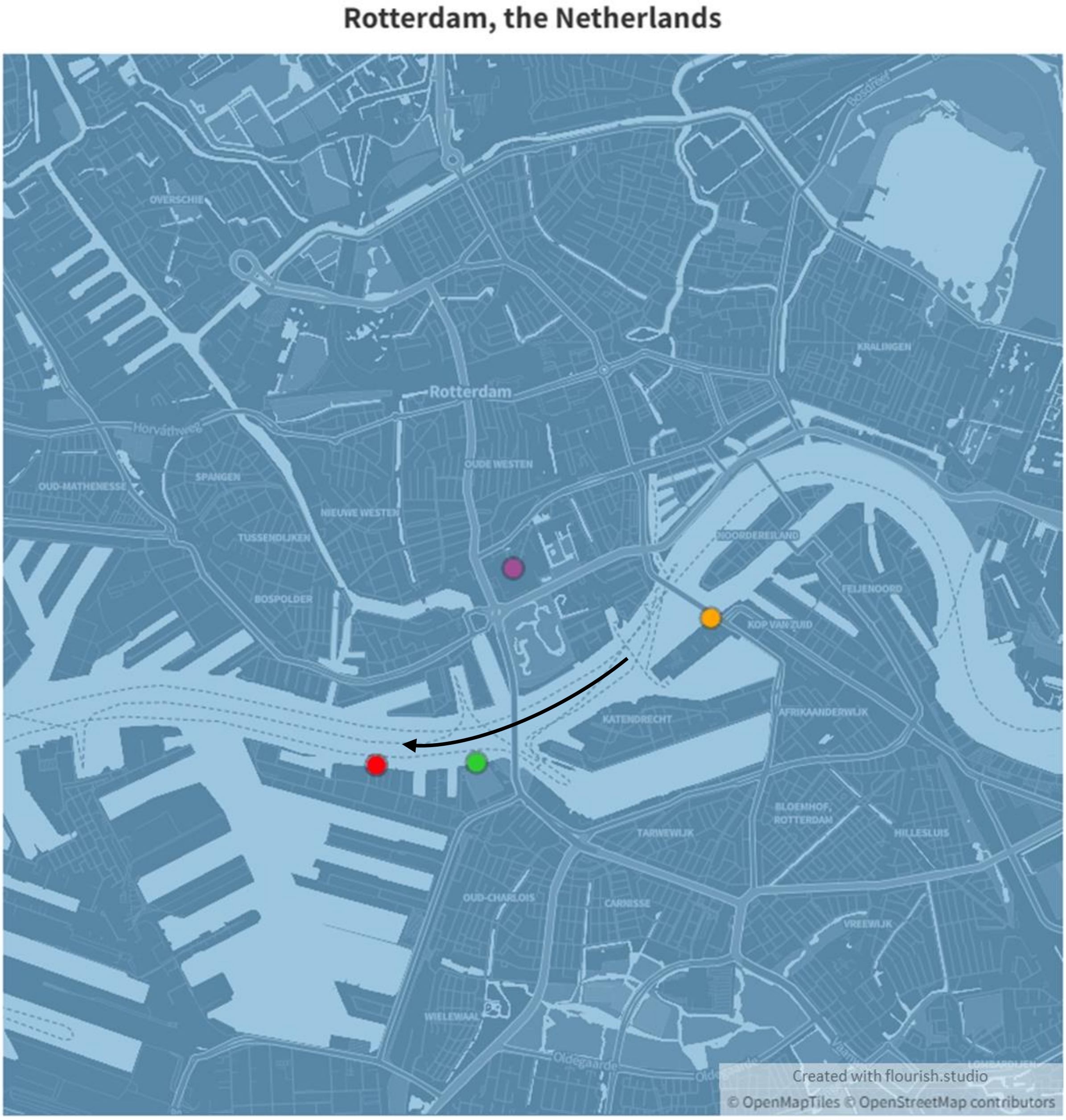

The purple dot indicates the Erasmus MC University Medical Center; the green dot indicates the municipal WWTP (mWWTP), including its outlet into the 'Nieuwe Maas' river; the yellow dot indicates the sampling site river upstream of the mWWTP; the red dot indicates the sampling site river downstream of the mWWTP. The black arrow shows the direction of the current, which is mostly from the East to the West.

Figure S5 Water sampling sites outside the hospital in Rome, Italy

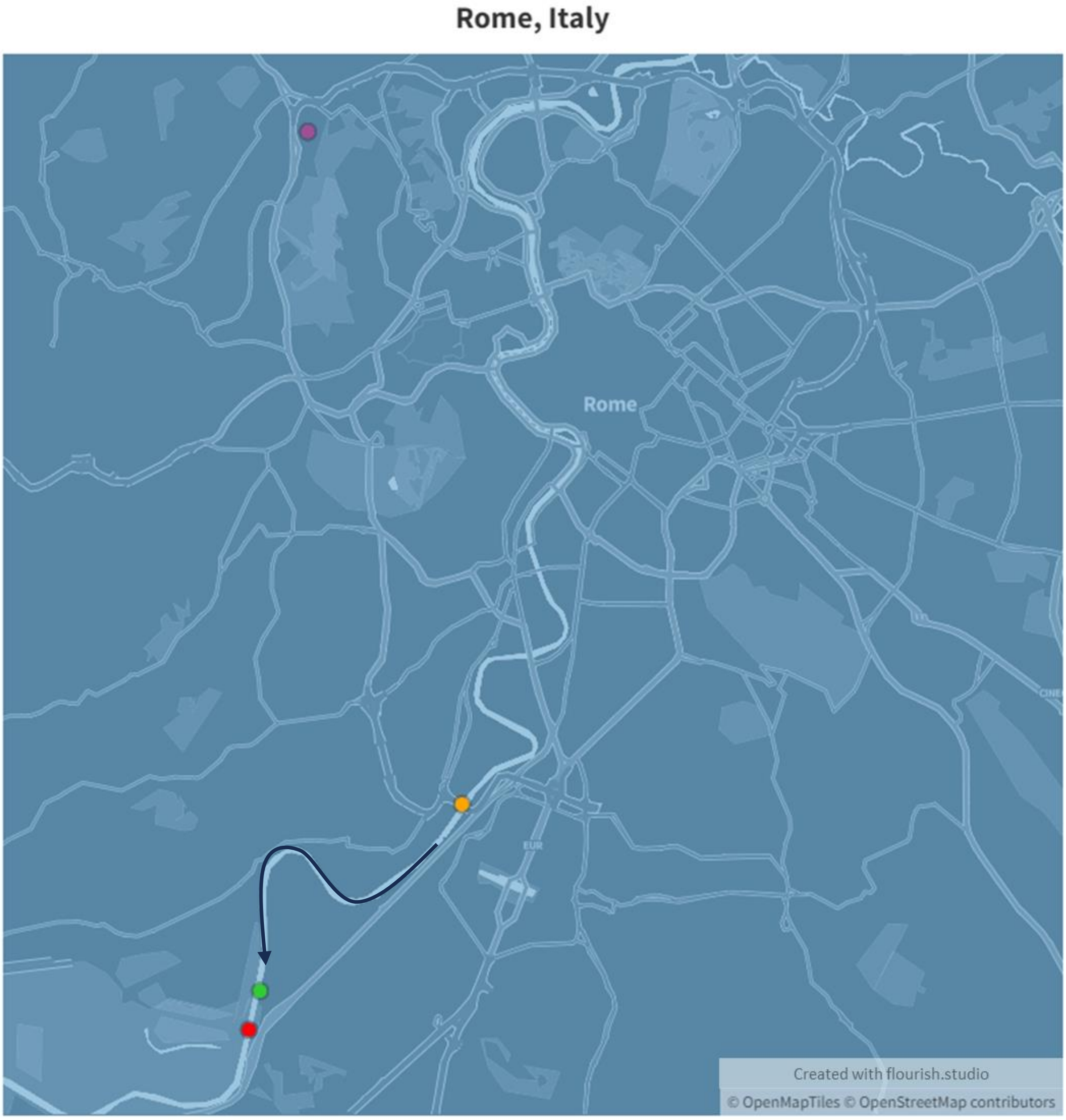

The purple dot indicates the Fondazione Policlinico Universitario Agostino Gemelli IRCCS; the green dot indicates the municipal WWTP (mWWTP), including its outlet into the 'Tevere' river; the yellow dot indicates the sampling site river upstream of the mWWTP; the red dot indicates the sampling site river downstream of the mWWTP. The black arrow shows the direction of the current, which is from the North to the South.

Figure S6 Water sampling sites outside the hospital in Jakarta, Indonesia

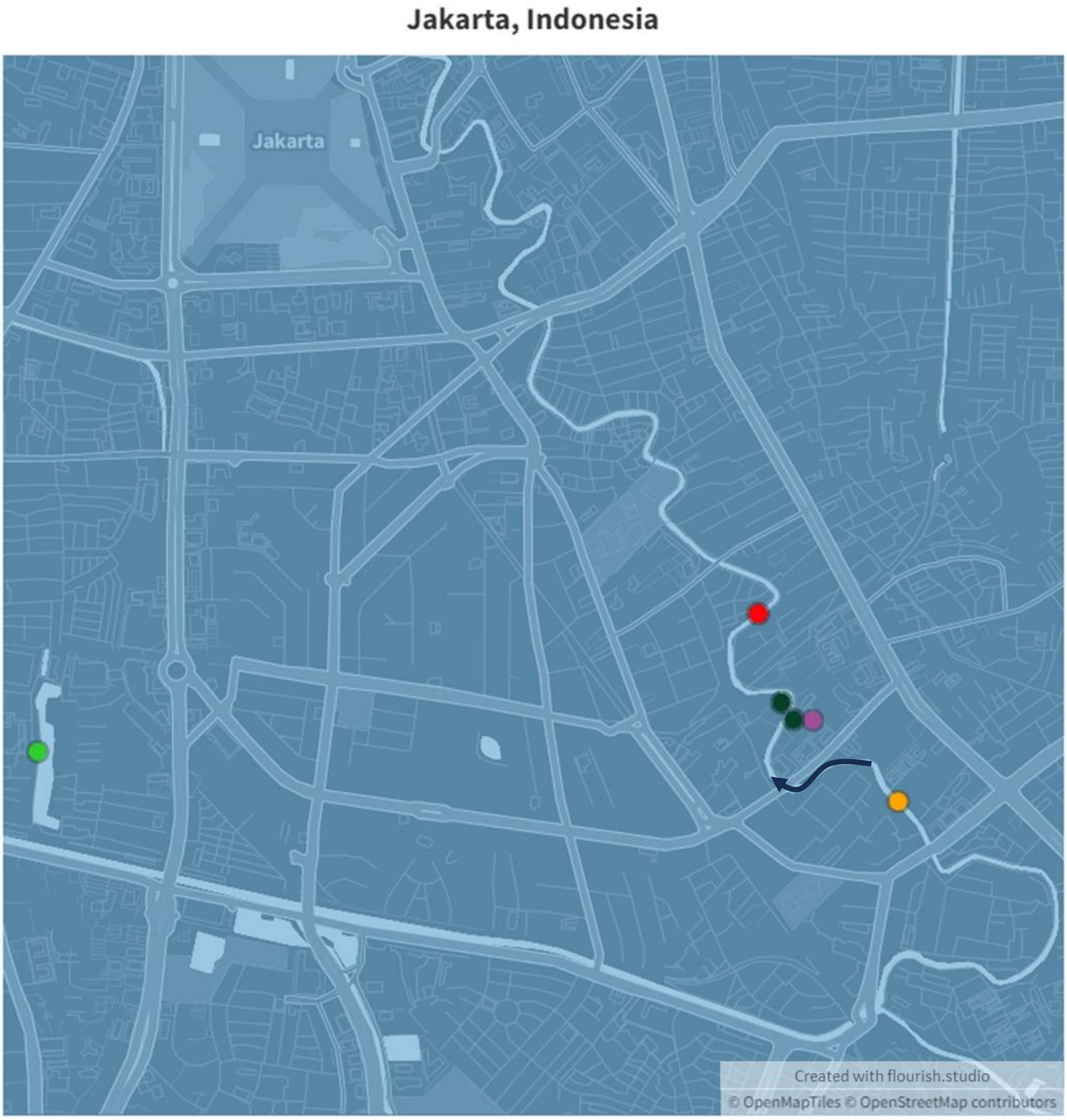

The purple dot indicates the Dr. Cipto Mangunkusumo General Hospital; the bright green dot indicates the municipal wastewater treatment plant (mWWTP); the dark green dots indicate the two hospital wastewater treatment plants (hWWTPs), including their outlets into the 'Ciliwung' river; the yellow dot indicates the sampling site river upstream of the hWWTPs; the red dot indicates the sampling site river downstream of the hWWTPs. The black arrow shows the direction of the current, which is from the Southeast to the Northwest. Groundwater samples will be collected close to the sampling site river downstream.

Figure S7 Wastewater treatment system of the hospital in Rotterdam, the Netherlands

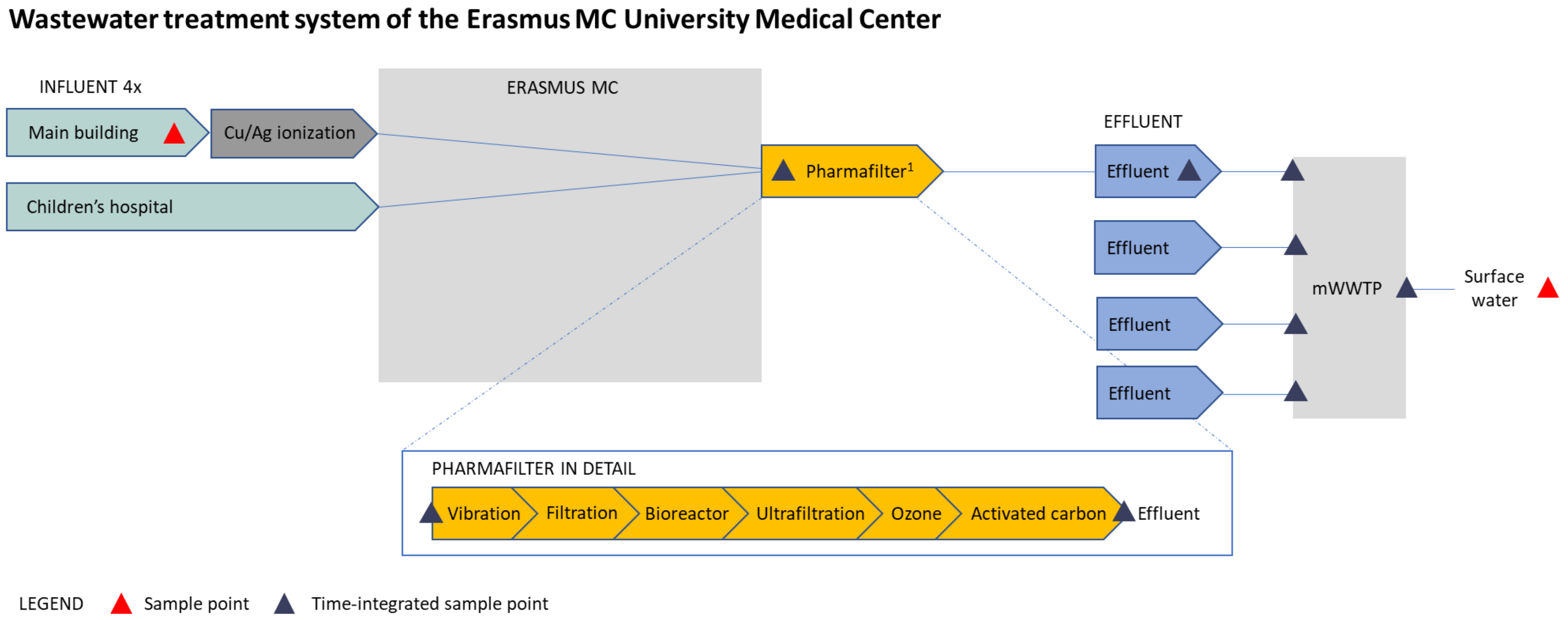

mWWTP municipal wastewater treatment plant

[1] The Erasmus MC University Medical Center has an advanced on-site hospital wastewater treatment system called Pharmafilter, similar to the Pharmafilter installation described by Paulus et al. (1).

Reference

1. Paulus GK, Hornstra LM, Alygizakis N, Slobodnik J, Thomaidis N, Medema G. The impact of on-site hospital wastewater treatment on the downstream communal wastewater system in terms of antibiotics and antibiotic resistance genes. Int J Hyg Environ Health. 2019;222(4):635-44.

Figure S8 Wastewater treatment system of the hospital in Rome, Italy

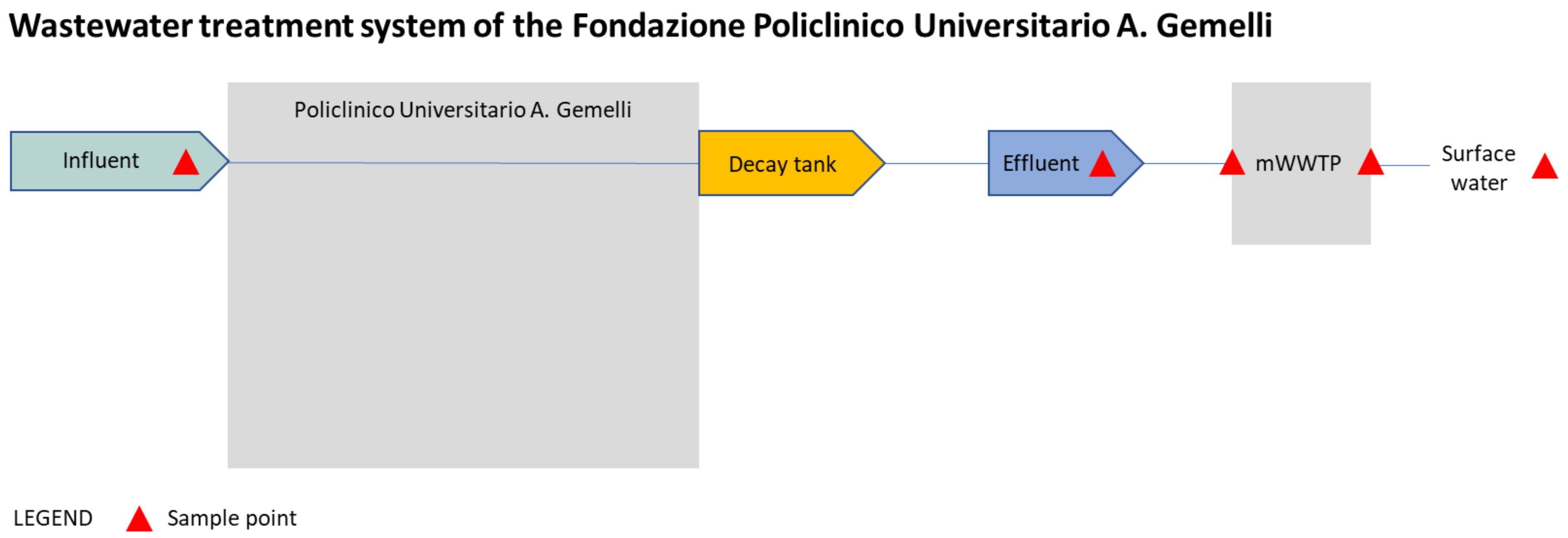

mWWTP municipal wastewater treatment plant

Figure S9 Wastewater treatment system of the hospital in Jakarta, Indonesia

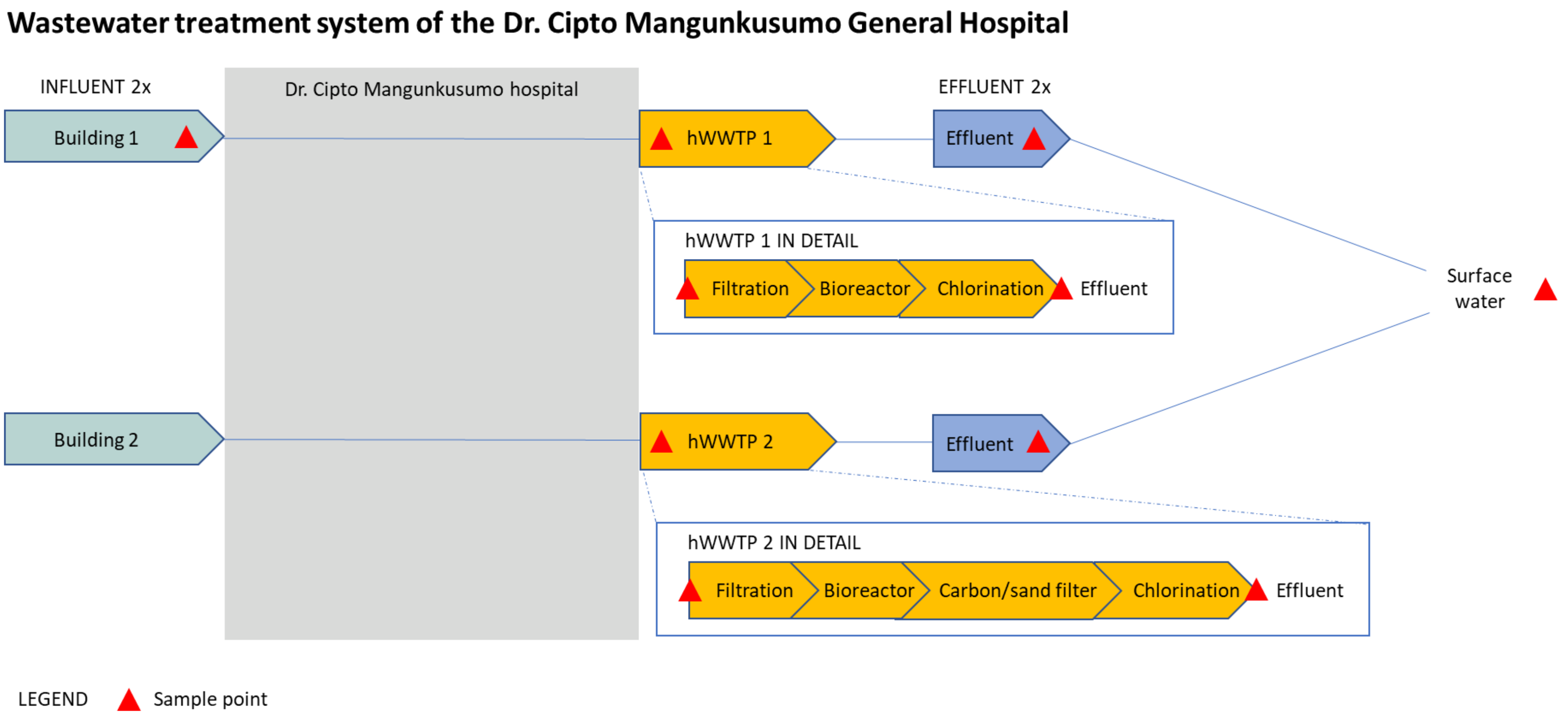

hWWTP hospital wastewater treatment plant

Table S2 Filtered volumes per sampling site for the isolation and quantification of CRPA

	Rotterdam, the Netherlands	Rome, Italy	Jakarta, Indonesia
**Hospital (drinking) water inlet**	500 mL<br>1000 mL<br>2 x 2000 mL	1 mL<br>10 mL<br>100 mL	10 mL<br>100 mL<br>500 mL<br>500 mL
**Influent of hWWTP**	0.001 mL<br>0.01 mL<br>0.1 mL<br>1 mL<br>10 mL	N/A	0.01mL<br>0.1 mL<br>1 mL<br>10 mL
**Effluent of hWWTP**	0.001 mL<br>0.01 mL<br>0.1 mL<br>1 mL<br>10 mL	N/A	0.01 mL<br>0.1 mL<br>1 mL<br>10 mL
**Untreated hospital wastewater**	N/A	1 mL<br>10 mL<br>100 mL	N/A
**Influent of mWWTP**	0.01 mL<br>0.1 mL<br>1 mL<br>10 mL<br>30 mL	1 mL<br>10 mL<br>100 mL	0.01 mL<br>0.1 mL<br>1 mL<br>10 mL
**Effluent of mWWTP**	0.01 mL<br>0.1 mL<br>1 mL<br>10 mL<br>100 mL	1 mL<br>10 mL<br>100 mL	0.01 mL<br>0.1 mL<br>1 mL<br>10 mL
**River upstream**	100 mL<br>2x 500 mL<br>2000 mL	1 mL<br>10 mL<br>100 mL	0.01mL<br>0.1 mL<br>1 mL<br>10 mL<br>100 mL
**River in proximity of hWWTP outlet**	N/A	N/A	0.01 mL<br>0.1 mL<br>1 mL<br>10 mL<br>100 mL
**River downstream**	100 mL<br>2x 500 mL<br>2000	1 mL<br>10 mL<br>100 mL	0.01 mL<br>0.1 mL<br>1 mL<br>10 mL<br>100 mL

**Community ground water**	N/A	N/A	10 mL<br>100 mL<br>500 mL<br>500 mL

hWWTP hospital wastewater treatment plant, *mWWTP* municipal wastewater treatment plant, *N/A* not applicable

Table S3 Number of colonies picked based on the observed number of colony morphologies

	**24 hours**	**48 hours (no colonies found after 24 hours)**	**48 hours (if colonies found after 24 hours)**
**1 morphology**	5 colonies	5 colonies	2 colonies (if different from 24 hours morphologies)
**2 morphologies**	3 colonies	3 colonies	2 colonies (if different from 24 hours morphologies)
**3 or more morphologies**	2 colonies	2 colonies	2 colonies (if different from 24 hours morphologies)
