# Supplementary material for: Sources and Transmission Routes of Carbapenem-Resistant Pseudomonas aeruginosa: Study Design and Methodology of the SAMPAN Study
Source: Antibiotics (Basel). 2025 Jan 15;14(1):94. doi: 10.3390/antibiotics14010094 (PMC11763197; doi:10.3390/antibiotics14010094)
Supplement: Supplementary file 1 [file antibiotics-14-00094-s001.zip › Supplementary file S2.pdf]

Supplementary file S2: Questionnaire international SAMPAN study

Your answers will remain anonymous. We do not save your name with your answers.

Filling in the questionnaire takes approximately 10 – 15 minutes.

Date of questionnaire completion: ……..

Part 1: General questions

Question 1
What is your gender?
O Female
O Male
O Other

Question 2
What is your age?
……………………………………………………..

Question 3
How do you live?
O Independent, with …… other persons.
O In a healthcare facility. Examples of healthcare facilities are: nursing homes, rehabilitation centers, residential centers for physically and mentally disabled individuals, retirement homes and psychiatry. Please write down the type of healthcare facility:
……………………………………………………..

Question 4
Do you have a job (paid or voluntary)?
O Yes, what is your profession? (*Please write down as specific as possible: for example, not a farmer, but farmer on a pig farm; for example not a civil servant, but a police officer)*
………..
O No

Question 5
Are you in contact with water for **more than 1 hour per day** during your work? (*e.g. swim instructor, fisherman*)
O Yes, please explain:………………………………………………………………………………………
O No
O Not applicable

Question 6
Do you come in contact with animals **more than 3 times a week, for more than 1 hour per day**?
(For example with dogs, cats, pigs, cows, poultry)
O Yes, with: …………………………………………………………………………………………………….
O No

Part 2: Questions about your health

Question 7
Did you visit (or were visited by) a professional in manicures and/or pedicures in the **last 12 months**?
O No
O Manicures
O Pedicures
O Both

Question 8
Do you wear contact lenses?
O Yes
O No

Question 9
Do you smoke?
O Yes. How many cigarettes do you smoke per day?……………………………………………………..
O No

Question 10
Do you have a roommate that smokes inside?
O Yes
O No

Question 11
Do you drink alcohol?
O Yes, every day.
O Yes, sometimes.
O Never

Question 12
Do you have a lung disease?
O Yes, which disease?………………………………………………………………………………………..
O No

Question 13
Is your gallbladder removed or do you have a gallbladder disease?
O Yes, it is removed.
O Yes, which disease? ……………………………………………………………………………………….
O No
O I don't know

Question 14

Have you taken antibiotics in the last **3 months**?

For example: Amoxicillin, Amoxicillin/Clavulanic Acid (Augmentin), Ciprofloxacin (Ciproxin), Nitrofurantoin (Furabid), Doxycycline, Claritromycin (Klacid), Azitromycin (Zithromax), Trimethoprim, Cotrimoxazol (Bactrimel).

O Yes. *In the table you can fill in which antibiotics, when and for how long, and how you were given them.*

O No

O I don’t know

Name antibiotics*	When and how long?**	How?***

** *For example: January 2021, 1 week.*

*** *For example: Tablets, intravenous treatment, ointment, liquid, eye drops, ear drops.*

Question 15
Have you been admitted to a national hospital in the last **12 months**? (*with admission we mean at least 1 or more overnight stays in the hospital)*
O Yes → In the table you can fill in when and for how long you were admitted.
O No → Go to question 16.
O I don't know → Go to question 16.

Date of admission	Number of days admitted

At Question 15 'Yes':
Question 15A
Did you shower **during your hospital stay**?
O Yes
O No
O I don't know

At Question 15 'Yes':
Question 15B
Did you use the sink in the room or bathroom **during your hospital stay**? (*For example to wash your hands*)
O Yes
O No
O I don't know

Question 16
Do you have any of the following medical devices? *(multiple answers possible)*
O Urinary catheter
O Drain, where do you have this:……………………………………………………………………….
O Tracheostomy
O None

Part 3: Questions about travelling

Question 17

Have you travelled abroad in the past **12 months**?

O Yes → Go to Question 18

O No → Go to Part 4

Question 18

How many holidays/travels have you made in the past **12 months**, which countries have you visited, and what was the departure and the return date?

- If you have visited several countries during one holiday/travel, this counts as one holiday/travel.
- If you don't remember the exact departure and return dates, you can also write down the number of days.

Nr.	Country	Departure Date	Return Date	Kind of travel*
1				
2				
3				
4				
5				

**Kind of travel: for example backpacking, group travel, beach holiday, city trip, business trip, visiting family/friends, all-inclusive trip.*

Question 19

Have you been hospitalized **during your holiday/travel**? (*with admission we mean at least 1 or more overnight stays in the hospital)*

O Yes, during holiday/travel number(s)………., number of days admitted to the hospital: ..……………………………………………………………………………………………………….

O No → Go to Part 4.

O I don't know → Go to Part 4.

At Question 19 ‘yes’:

Question 19A

Did you shower **during your stay** in a foreign hospital?

O Yes
O No
O I don’t know

At Question 19 ‘Yes’:

Question 19B

Did you use the sink in the room or bathroom **during your stay** in a foreign hospital? (*For example to wash your hands*)

O Yes
O No
O I don’t know

<u>Part 4: Questions about contact with water</u>

Question 20

What type of water do you usually use for drinking?

O Commercial drinking water (water packaged in bottles or packs)
O Boiled water
O Filtered water
O Tap water
O Government’s water
O Pump water
O Well water
O River water
O Other, namely:…………………………………………………………………………………………….

Question 21

Do you treat your water in any way to make it safer to drink?

O No
O Yes, I filter the water
O Yes, I boil the water
O Other, namely:…………………………………………………………………………………………….

Question 22

What type of water do you usually use for preparing food?

O Commercial drinking water (water packaged in bottles or packs)
O Boiled water
O Filtered water
O Tap water
O Government's water
O Pump water
O Well water
O River water
O Other, namely:…………………………………………………………………………………………….

Question 23

Where do you mostly get your food for each meal? (*please place one X in the table for each meal during the day)*

Meal	Cook at home	Restaurant	Office/school catering	Warung/ Kantin
**Breakfast**				
**Lunch**				
**Dinner**				
**Snack(s)**				

Question 24

What type of water do you use for household activities? (*For example taking a shower, washing)*

O Commercial drinking water (water packaged in bottles or packs)
O Boiled water
O Filtered water
O Tap water
O Government's water
O Pump water
O Well water
O River water
O Other, namely:…………………………………………………………………………………………….

Question 25

Do you usually wash yourself in the shower or bath?

O Shower
O Bath
O Bak gayung

Question 26

Approximately how many times a week do you shower or take a bath?

Per day:...………………………………...

Per week:…………………………………

Question 27
Does your household have its own toilet facility?
O Yes
O No

Question 28
What kind of toilet facility do members of your household usually use?
A. Flush/pour flush to:
O Piped sewer system
O Septic tank
O Pit latrine
O Elsewhere, namely………………………………………………………………………………………
O I don’t know

B. Type of toilet:
O Western toilet (standing on the floor/hanging on the wall) with lid
O Western toilet (standing on the floor/hanging on the wall) without lid
O Chemical toilet
O Bidet
O Ventilated improved pit latrine (VIP)
O Pit latrine with slab
O Pit latrine without slab/open pit
O Composting toilet
O Bucket
O Hanging toilet/hanging latrine
O No facilities or bush or field
O Other, please specify: ……………………………………………………………………………..

Question 29
Have you suffered from flooding in your home in the **last 12 months**? *(multiple answers possible)*
O Yes, due to rain
O Yes, due to a burst water pipe
O Yes, due to an overflow of the river
O Yes, due to:…………………………………………………………………………………………..
O No

Question 30
Where do you wash most of your clothes? (*choose one answer*)
O Hand wash at home
O Hand wash at the laundry worker – separate from clothes of other households
O Hand wash at the laundry worker – mixed with clothes of other households
O Hand wash at the laundry worker – unknown whether mixed or separate
O A washing machine at home
O Self-service laundry/Laundromat
O Pick-up laundry service - separate from clothes of other households
O Pick-up laundry service - mixed with clothes of other households
O Pick-up laundry service - unknown whether mixed or separate
O A river

Question 31
Do you use detergent or soap to wash your clothes?
O Yes
O No

Question 32
How warm do you wash your towels?
O Lower than 60 degrees Celsius
O 60 degrees Celsius or higher
O I don't know

Question 33
Have you visited a public swimming pool in the **last 12 months**?
O Yes
O No

Question 34
Have you been to a sauna in the **last 12 months**?
O Yes
O No

Question 35
Do you practice any water sports? (*For example swimming, water skiing)*
O Yes
O No

Question 36
Did you swim in open water in nature in the **last 12 months**? *(For example the sea, a lake, a river)*
O Yes, namely:…………………………………………………………………………………………………
O No

Question 37
Do you do hobby fishing?
O Yes
O No

Part 5: Finally

We would like to point out that by returning the questionnaire, you give permission that your answers will be used for this study.

Thank you very much for completing this questionnaire!
